# Genomic surveillance of SARS-CoV-2 by sequencing the RBD region using Sanger sequencing from North Kerala

**DOI:** 10.3389/fpubh.2022.974667

**Published:** 2022-08-25

**Authors:** Dhananjayan Dhanasooraj, Prasanth Viswanathan, Shammy Saphia, Beena Philomina Jose, Fairoz Cheriyalingal Parambath, Saritha Sivadas, N. P. Akash, T. V. Vimisha, Priyanka Raveendranadhan Nair, Anuja Mohan, Nimin Hafeez, Jayesh Kumar Poovullathi, Shameer Vadekkandiyil, Sajeeth Kumar Keriyatt Govindan, Rajan Khobragade, KP Aravindan, Chandni Radhakrishnan

**Affiliations:** ^1^Multidisciplinary Research Unit, Government Medical College, Kozhikode, Kerala, India; ^2^Virus Research and Diagnostic Laboratory, Government Medical College, Kozhikode, Kerala, India; ^3^Department of Microbiology, Government Medical College, Kozhikode, Kerala, India; ^4^Department of General Medicine, Government Medical College, Kozhikode, Kerala, India; ^5^Department of Health and Family Welfare, Government of Kerala, Thiruvananthapuram, India; ^6^MVR Cancer Hospital, Kozhikode, Kerala, India; ^7^Department of Emergency Medicine, Government Medical College, Kozhikode, Kerala, India

**Keywords:** SARS-CoV-2, Receptor Binding Domain, Sanger sequencing, spike gene sequencing, Kerala, genomic surveillance

## Abstract

Next Generation Sequencing (NGS) is the gold standard for the detection of new variants of SARS-CoV-2 including those which have immune escape properties, high infectivity, and variable severity. This test is helpful in genomic surveillance, for planning appropriate and timely public health interventions. But labs with NGS facilities are not available in small or medium research settings due to the high cost of setting up such a facility. Transportation of samples from many places to few centers for NGS testing also produces delays due to transportation and sample overload leading in turn to delays in patient management and community interventions. This becomes more important for patients traveling from hotspot regions or those suspected of harboring a new variant. Another major issue is the high cost of NGS-based tests. Thus, it may not be a good option for an economically viable surveillance program requiring immediate result generation and patient follow-up. The current study used a cost-effective facility which can be set up in a common research lab and which is replicable in similar centers with expertise in Sanger nucleotide sequencing. More samples can be processed at a time and can generate the results in a maximum of 2 days (1 day for a 24 h working lab). We analyzed the nucleotide sequence of the Receptor Binding Domain (RBD) region of SARS-CoV-2 by the Sanger sequencing using in-house developed methods. The SARS-CoV-2 variant surveillance was done during the period of March 2021 to May 2022 in the Northern region of Kerala, a state in India with a population of 36.4 million, for implementing appropriate timely interventions. Our findings broadly agree with those from elsewhere in India and other countries during the period.

## Introduction

The virus responsible for COVID-19 was initially identified by the use of an unbiased sequencing from airway epithelial cells samples of patients (1). The Coronaviridae Study Group (CSG) of the International Committee on Taxonomy of Viruses recognized the virus as severe acute respiratory syndrome-related coronavirus, and designated it as SARS-CoV-2 (2). The full-length genomic RNA of SARS-CoV-2 is ~ 29,903 nt (3). Whole Genome Sequencing (WGS) by Next Generation Sequencing (NGS) is the gold standard for detection of new variants of SARS-CoV-2 which have immune escape properties, high infectivity, and variable severity. This is also helpful in genomic surveillance, and for planning appropriate and timely public health interventions. NGS facilities may not be available in small or medium research centers due to the high cost of setting up such a facility. Other main hurdles with NGS include delays in tracking variants from samples sent, especially if the centers are not easily connected. This may create delays in the implementation of necessary community interventions, especially if the patient has traveled from a hotspot region or is suspected of having a new variant. High cost of NGS-based tests precludes it as the option for an economically viable surveillance program which requires immediate result generation and patient follow-up. In the study reported here, we used a cost-effective facility which can be set up in a common research lab and the methods used can be replicated in centers with experience in nucleotide sequencing. Many samples can be processed at a time and can generate the results in a maximum of 2 days (1 day for a 24 h working lab).

Spike protein (S) of SARS-CoV-2 helps the virus to enter host cells, through transmembrane S glycoprotein, which forms homodimers protruding from the viral surface (4). The S protein is composed of 1,273 amino acids and consists of two subunits S1: involved in binding to the host cell receptor and S2: fusion of the viral and cellular membranes (5, 6). Major vaccines against SARS-CoV-2 as well as the first-generation antibody therapeutic agents at present are based on the Spike protein sequence of the Wuhan reference sequence (7–9). Hence mutations in the region may critically affect virus pathogenicity and vaccine efficacy (10–12). The S1 subunit contains a specific area namely the Receptor Binding Domain (RBD), which plays a major role in virus entry into the host cell (13, 14). RBD binds to the human angiotensin-converting enzyme 2 receptor (hACE2) and the proteolytic action of human proteases may also be an advantage for the virus entry into human cells (13). RBD is also a site of frequent mutation in different variants of the virus (15, 16) and mutations therein may be particularly important for viral pathogenicity and vaccine efficacy.

In the present study, we have analyzed the nucleotide sequence of the RBD region of SARS-CoV-2 by the Sanger sequencing with in-house developed methods. The program was intended for surveillance of variants in the Northern region of Kerala, a state in India with a population of 36.4 million.

## Methodology

This study was initiated in March 2021 and is still ongoing; here we report the data generated during the last 1 year. The details of samples collected from 2000 COVID positive patients received at Government Medical College, Kozhikode are presented in this article.

RNA extracted from nasopharyngeal/oropharyngeal swabs were used for the study. The initial samples (March 2021) were only from the Regional Viral Research and Diagnostic Laboratory (VRDL) in Govt Medical College, Kozhikode. As the program was supported by the Government of Kerala from April 2021, samples from other testing labs in Northern Kerala were also included in the study. As part of a research project and a program of the Government of Kerala State, extracted RNA samples were shipped for spike gene sequencing to the Multidisciplinary Research Unit (MRU) of Government Medical College, Kozhikode which is a facility supported by the Department of Health Research (DHR) & Indian Council of Medical Research (ICMR), Government of India. The period of sample collection was from March 2021 to May 2022. More than 2,000 samples were processed during this period. As there was a decline in the number of cases from October to December 2021 the program had to be temporarily paused, but the sequencing could be restarted when there was an increase in the number of cases in Kerala from January 2022 and is still ongoing at the facility.

In the present study, we identified variants of the virus by the nucleotide sequence of the RBD region of SARS-CoV-2 by the Sanger sequencing with in-house developed methods. This study was approved by the Institutional Ethical Committee of Government Medical College, Kozhikode.

Study samples were selected from subjects meeting the below inclusion criteria for the sample received for the first phase of the study (from March 2021 to Oct 2021).

1. Recent International Traveler.
2. Reinfection- Persistent infection cases (patients who have an RT PCR positive in ≥90 days after initial infection regardless of symptoms or those who have an RT PCR positive in ≥45–89 days after recovery from the initial infection and with new onset of symptoms).
3. Cluster of cases.
4. Samples from the High-test positivity ratio (TPR) area.
5. Vaccine escape mutants: Infection after receiving one or two doses of vaccination.
6. Patient with no comorbidity but admitted to ICU or critically ill with severe disease.

The criteria for the samples received during the second phase of the study (received starting from January 2022) was expanded into the following categories to include more samples to look for new variants.

1. Cat 1: All symptomatic [Influenza-like illness (ILI) symptoms] cases including health care workers and frontline workers.
2. Cat 2: All asymptomatic direct and high-risk contacts (contacts in family and workplace, elderly ≥ 65 of age.
3. Cat 3: All asymptomatic high-risk individuals.
4. Cat 4: All symptomatic (ILI symptoms) individuals with a history of international travel in the last 14 days.
5. Cat 5: All symptomatic (ILI symptoms) contacts of a laboratory confirmed case.
6. Cat 6: All symptomatic (ILI symptoms) health care workers/frontline workers involved in containment.
7. Cat 7: All symptomatic ILI cases among returnees and migrants within 7 days of illness.
8. Cat 8: All asymptomatic high-risk contacts (contacts in family and workplace, elderly ≥ 65 years of age).
9. Cat 9: All patients of Severe Acute Respiratory Infection (SARI).
10. Cat 10: All symptomatic (ILI symptoms) patients presenting in a healthcare setting.
11. Cat 11: Asymptomatic high-risk patients who are hospitalized or seeking immediate hospitalization.
12. Cat 12: Asymptomatic patients undergoing surgical/non-surgical invasive procedures (not to be tested).
13. Cat 13: All pregnant women in/near labor who are hospitalized for delivery.
14. Cat 17: All individuals who wish to get themselves tested.
15. Surveillance subgroup (SSGr) 6: Epidemiological Samples.
16. SSGr 8: Elderly at Community.
17. Others- as per the discretion of the clinician.

### RNA isolation

Viral RNA from throat/nasal or nasopharyngeal swabs of patients were extracted using commercially available extraction kits/automated extraction machines by different laboratories in Kerala as part of COVID-19 testing. The RNAs were tested for SARS-CoV-2 specific RT PCR for detection of the virus at VRDLs of Government Medical Colleges and other Government COVID testing laboratories. The RNA samples with a CT value of <30 was found to be successfully sequenced; hence were included in the study.

### PCR amplification of the RBD region of spike (S) gene

Viral RNA received were reverse transcribed and PCR amplified in a single step RT PCR using custom designed primers covering the RBD region of the virus. In brief, the RBD area specific single step RT-PCR was performed on viral RNA using PrimeScript™ kit (One Step RT-PCR Kit Ver.2, **Takara Bio Inc, Japan**) according to manufacturer's instructions on PCR machine (Mastercycler nexus gradient, **Eppendorf, Germany**). The reaction included the following steps, a reverse transcription step at 50°C for 30 min, followed an initial 2-min hold at 94°C, amplification of the RBD region by 30 cycles of denaturing at 94°C (30 s), annealing at 55°C (30 s), and extension at 72°C (1 min), with a final 5-min extension at 72°C and a 4°C hold. Two sets of primers (**Integrated DNA Technologies, US**) were designed for the procedure and used in such a way that if the initial PCR fails, the next set of primers would be used for amplification. The primer details are given in Table 1. The primer combination CVSP3F and CVSP4R resulted in a product without any non-specific amplification, hence the PCR product was treated with Exosap-IT (**Thermo Scientific, US**) and proceeded with a sequencing PCR reaction. While the second set of primers CVSB2F and CVSB2R, the product was with a non-specific amplification of different size, hence the PCR product was first run on an agarose gel and was gel eluted using a spin column method (NucleoSpin® Gel and PCR Clean-up kit, **Takara Bio Inc, Japan**).

**Table 1:** Details of the primers used in the study.

**Sl. No**.	**Name**	**Sequence**	**Amplicon size**
1	CVSP3F	TGTGCACTTGACCCTCTCTC	1,144 bp
2	CVSP4R	CGCATATACCTGCACCAATG	
3	CVSB2F	GTGAAGTTTTTAACGCCACCAGATTTGC	854 bp
4	CVSB2R	AGCAACAGGGACTTCTGTGC	
5	CVSP4F	CTATCAGGCCGGTAGCACAC	Sequencing only

### Nucleotide sequencing of the RBD region

The Exosap treated or gel eluted PCR products were used for bidirectional nucleotide sequencing by the Sanger method. In brief, the sequencing reactions were carried out on the above products using specific primers and the BigDye™ Terminator v3.1 Cycle Sequencing Kit (**Thermo Scientific, US**). The same primers were used for sequencing the PCR product resulting from the primer combination CVSP3F and CVSP4R. But a different primer, CVSP4F, was used as the forward primer for the PCR product resulting from CVSB2F and CVSB2R, as the primer CVSB2F was not good for the sequencing reaction.

The products resulting from bidirectional sequencing were treated with the BigDye XTerminator™ Purification Kit (**Thermo Fisher Scientific Inc, US**) and kept for capillary sequencing on the ABI 3500 Genetic analyzer (**Thermo Scientific, US**). The chromatogram files generated were analyzed for mutations and identification of variants.

### Analysis of sequence data and identification of virus variants

As we handled many samples at a time, the initial step in data analysis was to check for common mutations and categorize the variants based on the preliminary data. The Linux (Ubuntu) based mutation calling software, “**covid-spike-classification** (CSC)”, was used for the same (17). CSC is a script to call relevant SARS-CoV-2 spike protein mutations from Sanger sequencing data. The program works on the Bioconda environment, the command instructions can handle chromatogram files (.ab1) in compressed form and the output file generated is in .csv format. The result file contains details such as amino acid changes identified in respective positions of spike protein. The identification of other mutations, which are not listed by CSC, was done after using a webtool **Coronapp** (18). The program is a web application written in Shiny and it is able to annotate amino acid changes from user-provided sequence-files, hence all mutations are traced out and given as a .csv file. Coronapp requires sequence files in FASTA format to work properly. The identified mutations were also inspected manually on respective chromatograms. The virus variants were identified based on type of amino acid changes in the RBD region (Supplementary Table 1).

## Results

As the study was conducted in two phases, and selection criteria were modified in the second phase. The number of patients in each category in the first and second phases are given in Tables 2, 3 respectively. It can be seen that for the first phase (March 2021 to Oct 2021) the highest number of samples were from clusters and vaccine breakthrough Infection (infection after two doses of vaccination). This was followed by samples from the High TPR area and vaccine breakthrough after a single dose of vaccine. The criteria followed for the second phase of the study were broad categories as listed in the methods section. Even though the program was designed to cover the northern region of Kerala, most of the samples were mainly from three districts, namely Kozhikode, Malappuram and Thrissur.

**Table 2:** Details of samples collected during phase 1 (March 2021 to October 2021) which met the inclusion criteria.

**Sl. No**.	**Category (phase 1)**	**Number**	**Percentage (%)**
1	Cluster	281	40.03
2	High TPR area	143	20.37
3	Infection after Single dose vaccine	56	7.98
4	Infection after Two doses of vaccine	212	30.20
5	Patient with no co-morbidity in ICU	1	0.14
6	Reinfection	6	0.85
7	Traveler from abroad	3	0.43

**Table 3:** Details of samples collected during phase 2 (January 2022 to May 2022) which met the inclusion criteria.

**Sl. No**.	**Category (phase 2)**	**Number**	**Percentage (%)**
1	Cat 1: All symptomatic (ILI symptoms) cases including health care workers and frontline workers	107	15.09
2	Cat 2: Asymptomatic direct and high-risk contacts (contacts in family and workplace, elderly ≥ 65	124	17.49
3	Cat 3: Asymptomatic high-risk individuals	76	10.72
4	Cat 4: All symptomatic (ILI symptoms) individuals with history of international travel in the last 14 days	2	0.28
5	Cat 5: All symptomatic (ILI symptoms) contacts of a laboratory confirmed case	100	14.10
6	Cat 6: All symptomatic (ILI symptoms) health care workers/frontline workers involved in containment	12	1.69
7	Cat 7: All symptomatic ILI cases among returnees and migrants within 7 days of illness	3	0.42
8	Cat 8: Asymptomatic high-risk contacts (contacts in family and workplace, elderly ≥ 65 years of	51	7.19
9	Cat 9: All patients of Severe Acute Respiratory Infection (SARI)	4	0.56
10	Cat 10: All symptomatic (ILI symptoms) patients presenting in a healthcare setting	22	3.10
11	Cat 11: Asymptomatic high-risk patients who are hospitalized or seeking immediate hospitalization	8	1.13
12	Cat 12: Asymptomatic patients undergoing surgical/non-surgical invasive procedures (not to be test	20	2.82
13	Cat 13: All pregnant women in/near labor who are hospitalized for delivery	1	0.14
14	Cat 17: All individuals who wish to get themselves tested	150	21.16
15	Others/miscellaneous	29	4.09

During the second phase of the study, the highest cases were from Cat 17: All individuals who wish to get themselves tested (21.16%), followed (17.49%) by Cat 2: All asymptomatic direct and high-risk contacts (contacts in family and workplace, elderly ≥ 65) (Table 3).

During the early phase of the study, the majority of samples showed three amino acid changes in spike protein such as L452R, T478K and D614G. As the RBD region has L452R and T478K mutations, these are identified as Delta (B1.617.2). Very few samples also had an additional spike protein amino acid change Q613H along with L452R and T478K. During September 2021 we found a Delta sample with an additional mutation of N501Y, hence there was a confusion in classification based on the sequence of RBD. We informed the same to the Department of Health, Government of Kerala to follow up the sample, and also send this RNA sample to an outside lab for whole genome sequencing, as per direction from the Government of Kerala.

Amino acid changes such as N501Y and A570D were observed in rare cases. With the presence of N501Y and absence of any other mutations in the RBD area these were identified as Alpha variants (B.1.1.7). In the same period, we found a few samples with mutations resulting in amino acid changes such as K417N, E484K and N501Y that could be identified as Beta variants (B1.351). L452R and E484Q were found in one sample during the period and there were no other mutations in the area to consider as the Kappa variant (B1.617.1). In a few cases we found only D614G amino acid change. Two samples were without any mutations, which can be considered as the original strain in circulation during the period.

From January 2022 we started getting amino acid changes in the spike protein such as S371L, S373P, S375F, K417N, N440K, G446S, S477N, E484A, Q493R, G496S, Q498R, N501Y, Y505H, T547K, and D614G, as these mutations are feature of omicron variant (B.1.1.529) they were identified as such and reported. During the end of January, the relative frequency of samples with G446S, G496S and T547K amino acid changes decreased, and samples without G446S mutations became prominent during March and April of 2022. This was significant, denoting a change in the number of omicron subvariant BA1 to BA2 in the population.

By April- May 2022, major amino acid changes in received samples were S371F, T376A, R408S, K417N, N440K, S477N, T478K, E484A, Q493R, Q498R, N501Y, Y505H, and D614G in spike gene signifying the presence of BA2 (B.1.1.529, sub-variant BA2) as main variant. At the end of May 2022, a few sub-variants with K417T, in addition to the above mutations, appeared. At the same time a sample with Omicron-BA2 with amino acid changes in spike protein such as G339D, S371F, S373P, S375F, T376A, D405N, R408S, K417N, N440K, **L452M**, S477N, T478K, E484A, Q493R, Q498R, N501Y, Y505H, D614G, and H655Y also appeared. As the sample showed BA2 features with L452M change, this could be a Variant of Concern under Monitoring during the period (19).

**Figure 1:**
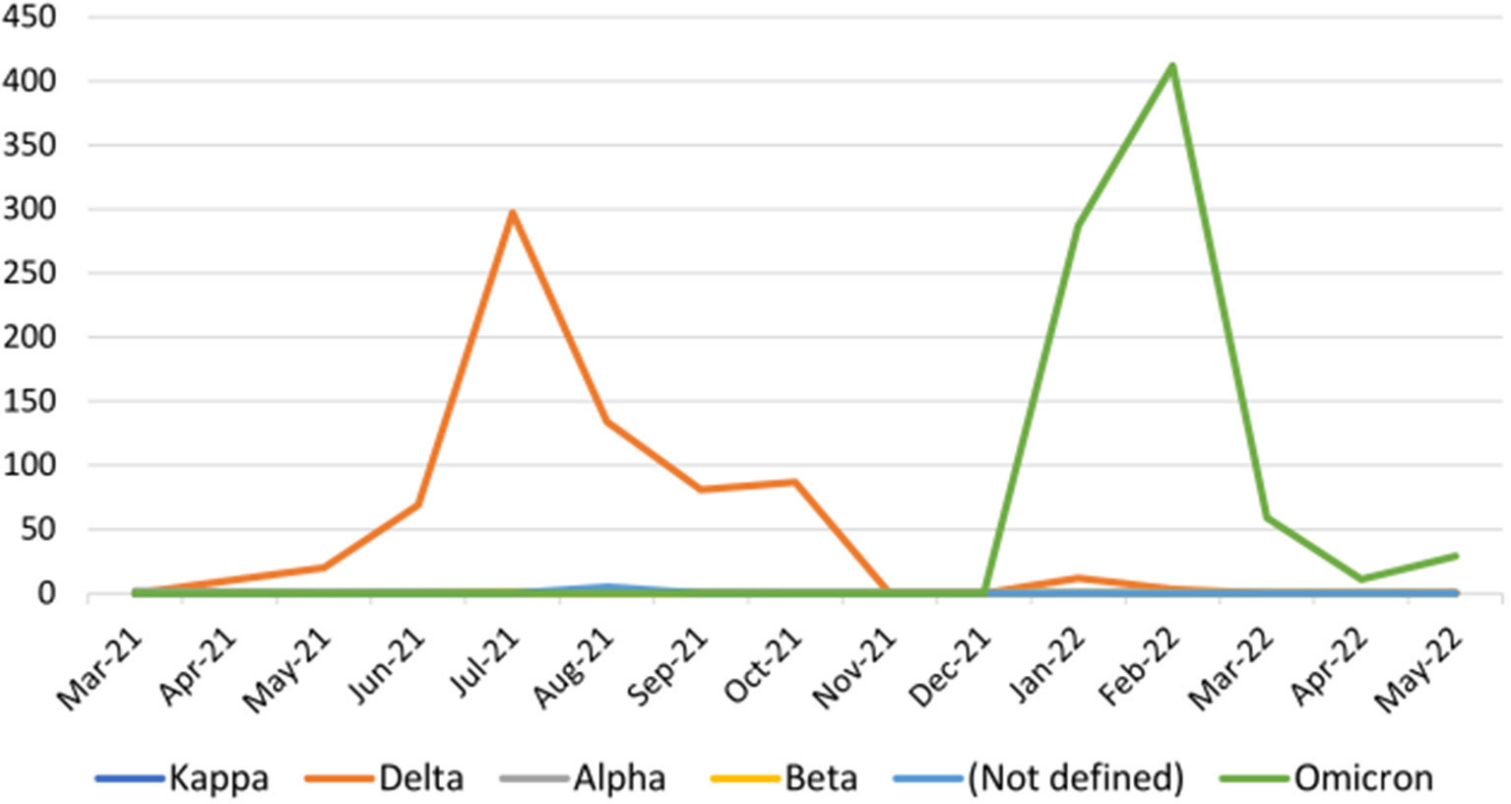
The variants detected per month during the study period (samples received from March 2021 to May 2022).

In the whole study period Delta and Omicron constituted the huge majority of samples, the others being obtained in negligibly small numbers (Table 4). On a timeline analysis, the Delta peaked in Kerala during the period of July to August 2021 and became almost rare by January 2022. Omicron reached its highest peak from January to February 2022 (Figure 1).

**Table 4:** Variants of SARS-CoV-2 identified in the study.

**Sl. No**.	**Variant**	**Number**	**Percentage (%)**
1	B1.1.7 (Alpha)	4	0.27
2	B1.351 (Beta)	1	0.07
3	B1.617.1 (Kappa)	1	0.07
4	B1.617.2 (Delta)	701	
5	Delta (unspecified)	2	
6	All Delta (Sl. No. 4 & Sl. No. 5)	703	46.59
7	Omicron	798	52.88
8	Not defined	5	0.33

50.5 % of the total RNA samples were from females. This was 47.7% for the Delta variant and 53.0% for Omicron.

**Figure 2:**
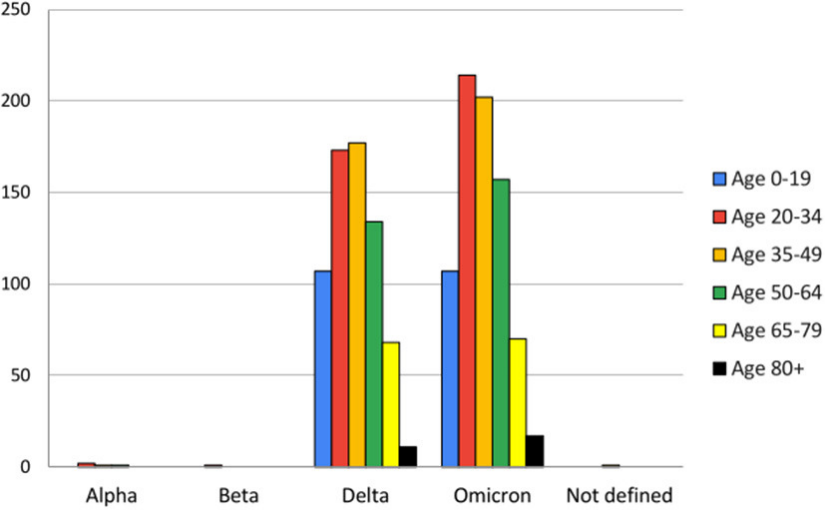
Age-wise analysis of variants observed during the study period (from March 2021 to May 2022).

The age-wise analysis of data showed that the 35–49 group was most affected by Delta and the 20–34 group by Omicron. Other groups followed an almost similar pattern of infection in other age groups except age group 80+ in which there was a slightly higher number of cases for omicron than Delta. The age groups 20–49 are the most infected groups by all variants in the present study (Figure 2).

92.6% of patients in phase 2 of the study were vaccinated; 83% by Covishield (**AstraZeneca, Serum Institute of India**) and 8.6% by Covaxin (**Bharat Biotech**).

## Discussion

Next Generation Sequencing (NGS) is the gold standard for the detection of new variants of SARS-CoV-2 which have immune escape properties, high infectivity and variable severity. In an earlier study, from Kerala, sequencing and analysis of SARS-CoV-2 isolates revealed unique patterns of the transmission (20). As mentioned earlier, due to the high cost and logistical problems in processing a large number of samples for surveillance by NGS, we have tried out a more feasible option using Sanger sequencing.

The present study was conducted in two phases, following the two peaks of disease in the state. The first phase was of 8-month duration, from March 2021 to Oct 2021, during which period Kerala faced severe outbreaks and even a brief lock-down was imposed to contain the virus spread (21, 22).

In the study, we used two sets of primers for one-step RT PCR; the second set intended to be used for amplification if the first set failed. But the first primer sets worked in around 90% of cases and the second sets were required only for the remaining samples.

During sequence data analysis to find the virus variants, the Linux (Ubuntu) based mutation calling software, COVID-spike-classification, was used. As the program is straightforward and lists out all the changes that match the available data, it can identify amino acid changes for different variants of SARS-CoV-2, reported for the virus during the period. The open-source program was updated frequently as and when novel variants reported. This may be helpful for a mass-scale screening of samples but may also miss some novel mutations if it happened to be other than the changes listed by the program. We used an additional webtool, Coronapp which required FASTA as input to work properly. As the program lists out all mutations but requires listing out all amino acid changes to find the variant, this can find novel variants if any arise. We used this tool to analyze sequence data if the first tool found any suspected combination or in case of the samples in which we suspected to have some different mutation as per clinical data or geographic data. Hence the second tool may not be a first preference for immediate result generation while working with many samples at a time.

We categorized virus variants as per Supplementary Table 1, so that variants of the SARS-CoV-2 can be classified by the presence of the signature mutations and resulting amino acid changes.

As per our data, the Delta variant was highest in number during this period. By the end of September 2021, the number of new cases decreased, hence the sequencing program got a temporary pause. With an increase in the number of new cases from January 2022 we could restart the program. Omicron became the major variant during this period, almost replacing other variants in this period; this was in full agreement with whole genome data available during the period (23, 24). We also found other variants such as Alpha and Beta with very low numbers of representation during the period. Our findings and its comparison with waves in other Indian States, mainly seen in cities such as Chennai, Mumbai, Delhi and other countries, showed a similar pattern (25–27). Region specific and time bound information is very important in utilizing the data on genomic sequences for tracking the infection and for timely interventions in required points (26, 28).

The mutations in omicron variant such as K417N, N440K, G446S, S477N, T478K, E484A, Q493K, G496S, Q498R, N501Y, and Y505H may help the strain for more effective attachment to ACE2 receptor (29). The method used in the present study can detect all these mutations by Sanger sequencing of a single PCR product. As the PCR primers used in the study covers the sequences encoding RBD and flanking region of SARS-CoV-2, detection of amino acid changes in this area can identify the sub-lineages such as BA.1, and BA.2 of omicron variant. Even though the method can identify the sub lineages of omicron such as BA.3, BA.4, and BA.5, proper differentiation between these lineages may be difficult as the area sequenced for the present study is limited for such information. In brief, with the present method we can identify the sub lineage as BA.1, BA.2 or as any from BA.3, BA.4, and BA.5.

It has been reported that variants contain mutations on RBD region such as K378N, P384S, R346S, P384L, R403K, R408I, I410V, K417N, K417T, K444N, L452R, L455F, A475S, V483A, E484Q, E484K,F486L, F490L, F490S, Q493H, Q493L, Q493R, S494P, G496S, N501T, and N501Y, and may make the virus more infectious, escape or weak vaccine mediated immunity (30). We found that more than 92% of the samples we received during the second phase were of vaccine breakthrough cases, and the presence of the above mutation on these samples confirms the observation. An earlier study reported breakthrough infections after fifteen days of post-second dose of vaccination and the genomic variants were analyzed in this paper (31).

The gender-wise analysis of infection cases reveal no significant difference between male and females in case of infection. The 35–49 age group, which includes the working group, seems to be most affected by the Delta variant. But this data is not from a representative cross section sample, and no specific inference can be made from this observation. This could also be due to the strict lockdown implemented by the Government of Kerala and only working people were exposed during the period (21, 22). The infection by the Omicron variant was higher for the age group 20–30, during the second phase of this study. During this phase there was no statewide lockdown and it was with relaxed rules regulating movement of people.

As the Government of India initiated mass vaccination from January 2021 and the major vaccine used was Covishield, this was also reflected in the data presented here (32). The data also revealed that more than 92% of patient samples during the second phase of this study were vaccine breakthrough infections, signifying the omicron variant, known to have less vaccine effectiveness, being predominant during the period.

The sanger sequence data in one of the samples showed that it was with mutations L452R and T478K (signature mutations in RBD region for Delta variant) and N501Y (of Alpha variant), hence variant identification of the same was not feasible by Sanger alone. We immediately informed the Health Department, Government of Kerala and requested the sample to be followed up, even as follow-up of all contacts and search for any outbreaks was also carried out. In parallel, we also sent the sample for whole genome sequencing at CSIR-IGIB, New Delhi and found that the same mutations existed in the strain and suggested that it would be classified as Delta since it had the entire complement of Delta mutations. In addition, the sample showed a mutation resulting in N501Y (which is also shared by the Alpha variant). This incident and follow-up actions illustrate how a monitoring program is practical and feasible in such situations in real life settings.

Approximately 25% of the total samples received failed to amplify during the initial PCR steps or sequencing steps, hence results for those are not included. The reason for failure could be improper cold chain maintenance during transportation, very low sample volume in received tubes, presence of samples with CT values of more than 30 (as the samples were received from different centers) or possible variation in primer-binding sites on both sets of primers in those samples.

The full procedure in the method chosen starting from Viral RNA including, one-step RT PCR, PCR product purification, clean up, bidirectional sequencing and result analysis can be completed in a day, in brief the different steps with adequate time for sample handling may require nearly 10 h. to complete, hence for a 24 h working laboratory the result can be generated even within 24 h of receiving samples.

## Conclusion

This study highlights the usefulness of Sanger sequencing of RBD region of SARS-CoV-2, by in-house developed methods for genomic surveillance of the virus. The study conducted during the period of March 2021 to May 2022 was aimed for surveillance of the virus spread in the Northern region of Kerala, a state in India with a population of 36.4 million. The results obtained showed similar patterns to the data from other Indian states, and also other countries during the same period. This study provides an overview of the data generated for a sufficiently longer period using Sanger sequencing and can be replicated as an economically viable option for genomic surveillance in other areas with similar technical expertise. High-cost tests such as NGS can be judiciously limited by such an approach.

## Data availability statement

The raw data supporting the conclusions of this article will be made available by the authors, without undue reservation.

## Ethics statement

The studies involving human participants were reviewed and approved by Institutional Ethical Committee of Government Medical College, Kozhikode (Sanction No. GMCKKDRP 2021/IEC/51 dated 12 March 2021). The patients/participants provided their written informed consent to participate in this study.

## Author contributions

DD designed the laboratory works, collected, analyzed and presented data, and wrote the manuscript. PV and SSa supervised the laboratory work related to the real time PCR and collection of RNA samples. BJ, FP, PN, AM, and NH did management of virus samples and collection of patient data. SSi, NA, TV, and PN did laboratory work related to the RNA samples. JP, SV, and SG did patient management and sample collection from patients. RK planned and implemented the program. KA initialized conceptualization of the idea, gave critical feedback, and wrote the manuscript. CR conceptualized the idea, provided feedback, did patient management, and wrote the manuscript. All authors contributed to the article and approved the submitted version.

## Funding

The initial fund for the project was through SBMR (State Board of Medical Research, Government of Kerala) and further by unrestricted support and supply of consumables from the Kerala Medical Services Corporation (KMSCL), Government of Kerala. The equipment and scientific support were through the Multidisciplinary Research Unit, a DHR-ICMR Government of India supported scheme at Government Medical College, Kozhikode.

## Conflict of interest

The authors declare that the research was conducted in the absence of any commercial or financial relationships that could be construed as a potential conflict of interest.

## Publisher's note

All claims expressed in this article are solely those of the authors and do not necessarily represent those of their affiliated organizations, or those of the publisher, the editors and the reviewers. Any product that may be evaluated in this article, or claim that may be made by its manufacturer, is not guaranteed or endorsed by the publisher.

## Supplementary material

Supplementary Table 1 Classification as per changes in the RBD region. Variants were classified by the presence of the signature amino acid changes resulting from mutations. The number shows the position of the amino acid and changes were denoted by “>” symbol. The amino acid changes which appear additionally than usual changes are shown in parentheses.

## References

[1] Zhu N Zhang D Wang W Li X Yang B Song J. A novel coronavirus from patients with pneumonia in China, 2019. N Engl J Med. (2020) 382:727–33. 10.1056/NEJMoa2001017 31978945 PMC7092803

[2] Gorbalenya AE Baker SC Baric RS de Groot RJ Drosten C Gulyaeva AA. The species Severe acute respiratory syndrome-related coronavirus : classifying 2019-nCoV and naming it SARS-CoV-2. Nat Microbiol. (2020) 5:536–44. 10.1038/s41564-020-0695-z 32123347 PMC7095448

[3] Kim D Lee JY Yang JS Kim JW Kim VN Chang H. The architecture of SARS-CoV-2 transcriptome. Cell. (2020) 181:914–21.e10. 10.1016/j.cell.2020.04.011 32330414 PMC7179501

[4] Walls AC Park YJ Tortorici MA Wall A McGuire AT Veesler D. Structure, function, and antigenicity of the SARS-CoV-2 spike glycoprotein. Cell. (2020) 181:281–92.e6. 10.1016/j.cell.2020.11.032 32155444 PMC7102599

[5] Pillay TS. Gene of the month: the 2019-nCoV/SARS-CoV-2 novel coronavirus spike protein. J Clin Pathol. (2020) 73:366–9. 10.1136/jclinpath-2020-206658 32376714

[6] Duan L Zheng Q Zhang H Niu Y Lou Y Wang H. The SARS-CoV-2 spike glycoprotein biosynthesis, structure, function, and antigenicity: implications for the design of spike-based vaccine immunogens. Front Immunol. (2020) 11:576622. 10.3389/fimmu.2020.576622 33117378 PMC7575906

[7] Du L He Y Zhou Y Liu S Zheng BJ Jiang S. The spike protein of SARS-CoV — a target for vaccine and therapeutic development. Nat Rev Microbiol. (2009) 7:226–36. 10.1038/nrmicro2090 19198616 PMC2750777

[8] Greaney AJ Starr TN Gilchuk P Zost SJ Binshtein E Loes AN. Complete mapping of mutations to the SARS-CoV-2 spike receptor-binding domain that escape antibody recognition. Cell Host Microbe. (2021) 29:44–57.e9. 10.1016/j.chom.2020.11.007 33259788 PMC7676316

[9] Xia X. Domains and functions of spike protein in SARS-CoV-2 in the context of vaccine design. Viruses. (2021) 13:109. 10.3390/v13010109 33466921 PMC7829931

[10] Korber B Fischer WM Gnanakaran S Yoon H Theiler J Abfalterer W. Tracking changes in SARS-CoV-2 spike: evidence that D614G increases infectivity of the COVID-19 virus. Cell. (2020) 182:812–27.e19. 10.1016/j.cell.2020.06.043 32697968 PMC7332439

[11] Plante JA Liu Y Liu J Xia H Johnson BA Lokugamage KG. Spike mutation D614G alters SARS-CoV-2 fitness. Nature. (2021) 592:116–21. 10.1038/s41586-020-2895-3 33106671 PMC8158177

[12] Huang Y Yang C Xu X Xu W Liu S. Structural and functional properties of SARS-CoV-2 spike protein: potential antivirus drug development for COVID-19. Acta Pharmacol Sin. (2020) 41:1141–9. 10.1038/s41401-020-0485-4 32747721 PMC7396720

[13] Shang J Wan Y Luo C Ye G Geng Q Auerbach A. Cell entry mechanisms of SARS-CoV-2. Proc Natl Acad Sci USA. (2020) 117:11727–34. 10.1073/pnas.2003138117 32376634 PMC7260975

[14] Ma B Zhang Z Li Y Lin X Gu N. Evaluation of interactions between SARS-CoV-2 RBD and full-length ACE2 with coarse-grained molecular dynamics simulations. J Chem Inf Model. (2022) 62:936–44. 10.1021/acs.jcim.1c01306 35147419

[15] Cao Y Yisimayi A Bai Y Huang W Li X Zhang Z. Humoral immune response to circulating SARS-CoV-2 variants elicited by inactivated and RBD-subunit vaccines. Cell Res. (2021) 31:732–41. 10.1038/s41422-021-00514-9 34021265 PMC8138844

[16] Sanches PRS Charlie-Silva I Braz HLB Bittar C Freitas Calmon M Rahal P.. Recent advances in SARS-CoV-2 Spike protein and RBD mutations comparison between new variants Alpha (B117, United Kingdom), Beta (B1351, South Africa), Gamma (P1, Brazil) and Delta (B16172, India). J Virus Erad. (2021) 7:100054. 10.1016/j.jve.2021.100054 34548928 PMC8443533

[17] Jørgensen TS Blin K Kuntke F Salling HK Michaelsen TY Albertsen M. A rapid, cost efficient and simple method to identify current SARS-CoV-2 variants of concern by Sanger sequencing part of the spike protein gene. MedRxiv. (2021). 10.1101/2021.03.27.21252266 PMC9642040 36368344

[18] Mercatelli D Triboli L Fornasari E Ray F Giorgi FM. Coronapp: a web application to annotate and monitor SARS-CoV-2 mutations. J Med Virol. (2021) 93:3238–45. 10.1002/jmv.26678 33205830 PMC7753722

[19] Tracking SARS-CoV-2 Variants. Available from: https://www.who.int/activities/tracking-SARS-CoV-2-variants (accessed June 13, 2022).

[20] Radhakrishnan C Divakar MK Jain A Viswanathan P Bhoyar RC Jolly B. Initial insights into the genetic epidemiology of SARS-CoV-2 isolates from Kerala suggest local spread from limited introductions. Front Genet. (2021) 12:630542. 10.3389/fgene.2021.630542 33815467 PMC8010186

[21] Saha J Mondal S Chouhan P. Spatial-temporal variations in community mobility during lockdown, unlock, and the second wave of COVID-19 in India: a data-based analysis using Google's community mobility reports. Spat Spatio Temporal Epidemiol. (2021) 39:100442. 10.1016/j.sste.2021.100442 34774257 PMC9760334

[22] Soni P. Effects of COVID-19 lockdown phases in India: an atmospheric perspective. Environ Dev Sustain. (2021) 23:12044–55. 10.1007/s10668-020-01156-4 33424429 PMC7785398

[23] Singh UB Deb S Chaudhry R Bala K Rani L Gupta R. SARS-CoV-2 Omicron variant wave in India: advent, phylogeny and evolution. bioRxiv. (2022). 10.1101/2022.05.14.491911

[24] Desingu PA Nagarajan K. SARS-CoV-2 Omicron variant is spreading in different parts of the world in three different trends. J Med Virol. (2022) 94:2354–6. 10.1002/jmv.27646 35112360 PMC9015426

[25] Singh UB Rophina M Chaudhry R Senthivel V Bala K Bhoyar RC. Variants of concern responsible for SARS-CoV-2 vaccine breakthrough infections from India. J Med Virol. (2022) 94:1696–700. 10.1002/jmv.27461 34786733 PMC8662202

[26] Tian D Sun Y Zhou J Ye Q. The global epidemic of SARS-CoV-2 variants and their mutational immune escape. J Med Virol. (2022) 94:847–57. 10.1002/jmv.27376 34609003 PMC8661756

[27] Pattabiraman C Prasad P George AK Sreenivas D Rasheed R Reddy NVK. Importation, circulation, and emergence of variants of SARS-CoV-2 in the South Indian state of Karnataka. Wellcome Open Res. (2022) 6:110. 10.12688/wellcomeopenres.16768.2 35243004 PMC8857524

[28] Maitra A Raghav S Dalal A Ali F Paynter VM Paul D. PAN-INDIA 1000 SARS-CoV-2 RNA genome sequencing reveals important insights into the outbreak. BioRxiv. (2020). 10.1101/2020.08.03.233718

[29] Jung C Kmiec D Koepke L Zech F Jacob T Sparrer KMJ. Omicron: what makes the latest SARS-CoV-2 variant of concern so concerning? J Virol. (2022) 96:e02077–21. 10.1128/jvi.02077-21 35225672 PMC8941872

[30] Wang R Chen J Gao K Wei GW. Vaccine-escape and fast-growing mutations in the United Kingdom, the United States, Singapore, Spain, India, and other COVID-19-devastated countries. Genomics. (2021) 113:2158–70. 10.1016/j.ygeno.2021.05.006 34004284 PMC8123493

[31] J BP Jolly B John N Bhoyar RC Majeed N Senthivel V. Genomic survey of SARS-CoV-2 vaccine breakthrough infections in healthcare workers from Kerala, India. J Infect. (2021) 83:237–79. 10.1016/j.jinf.2021.05.018 34044037 PMC8143909

[32] Choudhary OP Choudhary P Singh I. India's COVID-19 vaccination drive: key challenges and resolutions. Lancet Infect Dis. (2021) 21:1483–4. 10.1016/S1473-3099(21)00567-3 34529961 PMC8437681

