# Supplementary material for: Genomic surveillance of SARS-CoV-2 by sequencing the RBD region using Sanger sequencing from North Kerala
Source: Front Public Health. 2022 Aug 25;10:974667. doi: 10.3389/fpubh.2022.974667 (PMC9454329; doi:10.3389/fpubh.2022.974667)
Supplement: Supplementary Table 1 — Classification as per changes in the RBD region. Variants were classified by the presence of the signature amino acid changes resulting from mutations. The number shows the position of the amino acid and changes were denoted by “>” symbol. The amino acid changes which appear additionally than usual changes are shown in parentheses. [file Table_1.docx]

|  |  |  | **Amino acid position in Spike protein of SARS-COV-2** | | | | | | | | | | | | | | | | | | | | | |
| --- | --- | --- | --- | --- | --- | --- | --- | --- | --- | --- | --- | --- | --- | --- | --- | --- | --- | --- | --- | --- | --- | --- | --- | --- |
|  |  |  | **RBD region** | | | | | | | | | | | | | | | | | | |  |  |  |
| **Sl. No.** | **WHO Name** | **PANGO Name** |  | **346** | **408** | **417** | **440** | **446** | **449** | **450** | **452** | **477** | **478** | **484** | **486** | **490** | **493** | **496** | **498** | **501** | **505** | **547** | **570** | **614** |
| 1 | **Alpha** | B.1.1.7 |  |  |  |  |  |  |  |  |  |  |  |  |  |  |  |  |  | **N>Y** |  |  | **A>D** | **D>G** |
| 2 | **Beta** | B.1.351 |  |  |  | **K>N** |  |  |  |  |  |  |  | **E>K** |  |  |  |  |  | **N>Y** |  |  |  | **D>G** |
| 3 | **Gamma** | P.1 |  |  |  | **K>T** |  |  |  |  |  |  |  | **E>K** |  |  |  |  |  | **N>Y** |  |  |  | **D>G** |
| 4 | **Delta** | B.1.617.2 |  |  |  |  |  |  |  |  | **L>R** |  | **T>K** |  |  |  |  |  |  |  |  |  |  | **D>G** |
| 5 | **Epsilon** | B.1.427, B1.429 |  |  |  |  |  |  |  |  | **L>R** |  |  |  |  |  |  |  |  |  |  |  |  | **D>G** |
| 6 | **Zeta** | P.2 |  |  |  |  |  |  |  |  |  |  |  | **E>K** |  |  |  |  |  |  |  |  |  | **D>G** |
| 7 | **Eta** | B.1.525 |  |  |  |  |  |  |  |  |  |  |  | **E>K** |  |  |  |  |  |  |  |  |  | **D>G** |
| 8 | **Theta** | P.3 |  |  |  |  |  |  |  |  |  |  |  | **E>K** |  |  |  |  |  | **N>Y** |  |  |  | **D>G** |
| 9 | **Iota** | B.1.526 |  |  |  |  |  |  |  |  |  | **(S>N)** |  | **(E>K)** |  |  |  |  |  |  |  |  |  | **D>G** |
| 10 | **Kappa** | B.1.617.1 |  |  |  |  |  |  |  |  | **L>R** |  |  | **E>Q** |  |  |  |  |  |  |  |  |  | **D>G** |
| 11 | **Lambda** | C37 |  |  |  |  |  |  |  |  | **L>Q** |  |  |  |  | **F>S** |  |  |  |  |  |  |  | **D>G** |
| 12 | **Mu** | B.1.621 |  | **R>K** |  |  |  |  |  |  |  |  |  | **E>K** |  |  |  |  |  | **N>Y** |  |  |  | **D>G** |
| 13 |  | C.1.2. |  |  |  |  |  |  | **Y>H** |  |  |  |  | **E>K** |  |  |  |  |  | **N>Y** |  |  |  | **D>G** |
| 14 | **Omicron** | B.1.1.529 | **G339D, S371L, S371F, S373P, S375F, T376A** |  | **R>S** | **K>N** | **N>K** | **G>S** |  |  | **L>R** | **S>N** | **T>K** | **E>A** | **F>V** |  | **Q>R** | **G>S** | **Q>R** | **N>Y** | **Y>H** | **T>K** |  | **D>G** |

**Supplementary table 1**: Variants were classified by the presence of the signature amino acid changes resulting from mutations. The number shows the position of the amino acid and changes were denoted by ‘>’ symbol. The amino acid changes which appear additionally than usual changes are shown in parentheses.
